# Editorial: New molecular targets involved in lung adenocarcinoma

**DOI:** 10.3389/fendo.2023.1138849

**Published:** 2023-01-23

**Authors:** Xianquan Zhan, Na Li

**Affiliations:** Medical Science and Technology Innovation Center, Shandong Key Laboratory of Radiation Oncology, Shandong Cancer Hospital and Institute, Shandong First Medical University & Shandong Academy of Medical Sciences, Jinan, Shandong, China

**Keywords:** lung adenocarcinoma, lung cancer, treatment, molecular targets, multiomics, integrative omics, combination effects

Primary lung cancer (PLUC) has high morbidity and mortality, which seriously impacts human health. In terms of pathological histological changes, PLUC is classified into non-small cell lung cancer (NSCLC; 70-80%) and small cell lung cancer (SCLC; 20-30%) (1, 2). NSCLC includes lung adenocarcinoma (LUAD) and lung squamous carcinoma (LUSC). Compared to LUSC, LUAD is often diagnosed in non-smoker people, with significant differences in biological behavior, response to therapy, and prognosis (3, 4). It is reported that gender and hormonal status are significantly different between LUAD and LUSC. In recent years, the studies on the mutations of driver genes in NSCLC have produced a certain targeted therapies that improve effectively therapeutic strategies to significantly enhance the survival benefits of LUAD patients (5). However, the overall survival is still low for LUAD patients. It is urgently necessary to clarify in-depth the molecular mechanisms, identify novel molecular therapeutic targets, and construct much more effective diagnostic or prognostic biomarkers for LUAD patients because the lack of novel molecular targets and biomarkers is the bottleneck to discover new effective treatments.

It is well-known that LUAD is a multi-cause, multi-process, and multi-consequence malignant cancer with high heterogeneity, which is involved in a series of molecular changes in DNA, RNA, protein, and metabolite, and these molecules work in a mutually associated molecular system (6–8). Multiomics or integrative omics analyses are effective approaches to identify novel molecular therapeutic targets and diagnostic or prognostic biomarkers for LUAD patients in the framework of predictive, preventive, and personalized medicine (PPPM; 3P medicine) and precision medicine (PM) (9, 10). This present research topic focuses on, but is not limited to, the use of multiomics and integrative omics approaches to identify new driver genes, discover effective molecular therapeutic targets, construct diagnostic or prognostic models to predict, diagnose, and prognostically assess LUAD to improve the survival of patients.

The present issue contained eight highly focused articles: (i) The first article focused on the difference of cancer stem cells (CSCs) and cancer stemness between LUAD and LUSC, and established the corresponding stemness index (mRNAsi) to classify LUAD or LUSC into lower- and higher-mRNAsi subtypes, which were used to construct an eight-immune-related gene-signature prognostic model (CD1B, ANGPTL5, CNTFR, CD1E, CTSG, IL12B, IL2, and EDN3) for LUAD, and a five-immune-related gene-signature prognostic model (KLRC3, CCL1, KLRC1, CCL23, and KLRC4) for LUSC (Li et al.). These mRNAsi-related immune-related genes were potential biomarkers for LUAD and LUSC. (ii) The second article focused on a new long noncoding RNA (lncRNA) LINC00467 in LUAD, which found that hypomethylation and DNA copy number amplification upregulated LINC00467 expression in LUAD to associate with its distant metastasis, immune infiltration, and poor survival, and established the LINC00467-ceRNA network that included two microRNAs (hsa-miR-1225-5p, hsa-miR-575) and five mRNAs (BCL9, BARX2, KIAA1324, KCNK1, and TMEM182) in LUAD to reveal that BCL9 and BARX2 were potential prognostic biomarkers for LUAD patients (Wang et al.). (iii) The third article experimentally confirmed that the uptake of ^18^F-AlF-NOTAPRGD2 (^18^F-RGD) during positron emission tomography (PET) was negatively correlated with the expression of tumoral programmed death-ligand 1 (PD-L1) in NSCLC, and found that its maximum standard uptake value (SUVmax) was the best parameter to display PD-L1 expression, and 18F-RGD PET was useful to reflect the immune status of NSCLC (Wu et al.). (iv) The fourth article focused on LUAD cell A549-secreted exosomes that were released into tumor microenvironment (TME) to regulate TME cells. This study found that miR-181b-5p and let-7c-5p expressed in A549-cell-derived exosomes induced invasion, migration, and epithelial-mesenchymal transition (EMT) of human bronchial epithelial cells BEAS-2B through MAPK signaling and focal adhesion pathways, and that tumor exosomal miR-181b-5p and let-7c-5p were the early diagnosis biomarker for LUAD patients (Liu et al.). (v) The fifth article addressed the significant differences in clinical features between LUAD and LUSC, including multiple factors such as gender, drinker, smoker, pathological stage, fever, cough, sputum, bacterial and fungal infections, patchy shadows and ground-glass opacity in imaging films, and the levels of platelets, leukocytes, and creatinine, which revealed that LUSC was a more malignant type compared to LUAD (Wang et al.). (vi) The sixth article focused on cuproptosis in LUAD, which analyzed 36 cuproptosis genes and 385 cuproptosis-related genes (CRGs), and found 17 upregulated and 3 downregulated cuproptosis genes in LUAD; established a cuproptosis-related gene signature to calculate risk score for dividing LUAD patients into high- and low-risk subgroups to predict the overall survival of LUAD patients; and also identified CRGs GFRA3 and BARX1 as the sensitive target for LUAD cells to the cuproptosis (Chen et al.). (vii) The seventh article addressed the combined model of radiomics and clinical features to discriminate pneumonic-type mucinous adenocarcinoma (PTMA) from lobar pneumonia (LP) in 199 qualified patients, with a powerful discriminative ability - the highest AUC value 0.94 (95. 31% CI, 0.90–0.98) in the training cohort, and 0.91 (95% CI, 0.84–0.99) in the validation cohorts. It clearly demonstrated that the combined model based on radiomics signatures of CT images and clinical risk factors can differentiate PTMA from LP, and provide accurate therapy decision for clinicians (Ji et al.). (viii) The eighth article addressed endothelin-converting enzyme 2 (ECE2) and its co-expression genes in LUAD. This study found that ECE2 was correlated positively with m6A methylation-related genes (IGF2BP1, IGF2BP3, HNRNPC, and RBM1), while ECE2was correlated negatively with the levels of immune cells (M2 macrophages, CD4+ cells, B cells, dendritic cells, and neutrophils). These results clearly demonstrated that ECE2 was a prognostic biomarker for LUAD (Zhang et al.).

The overall analysis of those 8 articles clearly demonstrated that this present issue provided a spectrum of new molecular targets and risk factors for LUAD, including new driver genes and mutation genes, new risk factors in individual and combined effects, new diagnostic and prognostic models, new therapeutic targets, new molecular mechanisms and vital signaling pathways, and multi-site combination therapy targets and strategies. Multiomics or integrative omics, including genomics, transcriptomics, proteomics, proteoformics (11, 12), biomolecular modification omics, and bioinformatics (11, 12), played important roles to identify new molecular targets and risk factors, discover effective therapeutic targets and biomarkers, and establish reliable diagnostic and prognostic signature models for patient stratification, personalized prediction, diagnosis, and prognostic assessment in the framework of PPPM and PM in LUAD (6–10). We emphasize the important scientific merits of multiomics or integrative omics analyses in discovery of new molecular targets involved in LUAD. We recommend to strengthen the multiomics studies on discovery of new molecular targets in LUAD in future. Moreover, we recommend to combine multiple molecules, multiple risk factors, radiomics features, and even clinical characteristics to construct a comprehensive combination model for management of LUAD patients.

In summary, studies on new molecular targets involved in LUAD have obtained significant achievements as described above, which provides new clues and important data for future in-depth studies, and might be translated into clinical applications. However, these eight articles contained in this special issue are only a very small fraction of new molecular targets in LUAD. This research topic will serve as a spur to stimulate and encourage researchers to engage in the studies on new molecular targets involved in LUAD and improve the overall survival of LUAD patients in the framework of PPPM and PM.

## Author contributions

XZ conceived the concept, collected and analyzed the literature, designed, wrote and critically revised manuscript, and was responsible for its financial supports and the corresponding works. NL conceived the concept, analyzed the literature, and participated in writing. All authors approved the final manuscript.
